# Comparison of carbapenem-susceptible and carbapenem-resistant *Enterobacterales* at nine sites in the USA, 2013–2016: a resource for antimicrobial resistance investigators

**DOI:** 10.1099/mgen.0.001119

**Published:** 2023-11-21

**Authors:** Joseph D. Lutgring, Alyssa G. Kent, Jolene R. Bowers, Daniel E. Jasso-Selles, Valerie Albrecht, Valerie A. Stevens, Ashlyn Pfeiffer, Riley Barnes, David M. Engelthaler, J. Kristie Johnson, Amy S. Gargis, J. Kamile Rasheed, Brandi M. Limbago, Christopher A. Elkins, Maria Karlsson, Alison L. Halpin

**Affiliations:** ^1^​ Division of Healthcare Quality Promotion, National Center for Emerging and Zoonotic Infectious Diseases, Centers for Disease Control and Prevention, Atlanta, Georgia, USA; ^2^​ Goldbelt C6, LLC, Chesapeake, Virginia, USA; ^3^​ Pathogen and Microbiome Division, Translational Genomics Research Institute North, Flagstaff, Arizona, USA; ^4^​ Department of Pathology, University of Maryland School of Medicine, Baltimore, Maryland, USA; ^†^​ Present address: Office of the Director, National Center for Emerging and Zoonotic Infectious Diseases, Centers for Disease Control and Prevention, Atlanta, Georgia, USA; ^‡^​ Present address: Office of Science, National Center for Immunization and Respiratory Diseases, Centers for Disease Control and Prevention, Atlanta, Georgia, USA

**Keywords:** CRE, genomic sequencing, carbapenem-resistant, carbapenem-susceptible, carbapenem-resistant *Enterobacterales*, molecular epidemiology

## Abstract

Carbapenem-resistant *Enterobacterales* (CRE) are an urgent public health threat. Genomic sequencing is an important tool for investigating CRE. Through the Division of Healthcare Quality Promotion Sentinel Surveillance system, we collected CRE and carbapenem-susceptible *Enterobacterales* (CSE) from nine clinical laboratories in the USA from 2013 to 2016 and analysed both phenotypic and genomic sequencing data for 680 isolates. We describe the molecular epidemiology and antimicrobial susceptibility testing (AST) data of this collection of isolates. We also performed a phenotype–genotype correlation for the carbapenems and evaluated the presence of virulence genes in *Klebsiella pneumoniae* complex isolates. These AST and genomic sequencing data can be used to compare and contrast CRE and CSE at these sites and serve as a resource for the antimicrobial resistance research community.

## Significance as a BioResource to the community

This unique dataset of 680 isolates significantly bolsters publicly available data for public health, molecular epidemiology and molecular biology efforts. It comprises high-quality genomic data from carbapenem-susceptible *Enterobacterales* (CSE) and confirmed carbapenem-resistant *Enterobacterales* (CRE) identified through routine culture at nine clinical laboratories from across the USA. The CSE and CRE were collected contemporaneously and from the same catchments allowing for comparison of the strain dynamics of these populations. This dataset serves as a significant resource for further analyses – the genomic data are curated with reference antimicrobial susceptibility data for each isolate. In addition, these data may be valuable for improving our understanding of phylogenomics, the spread of carbapenem resistance and molecular epidemiology.

## Data Summary

Sequence reads were submitted to NCBI’s Sequence Read Archive (SRA) database under the BioProject PRJNA288601. Individual BioSample accessions can be found in File S1, available in the online version of this article. The authors confirm all supporting data, code and protocols have been provided within the article or through supplementary data files. This article contains data hosted by Microreact.

## Introduction

Carbapenem-resistant *Enterobacterales* (CRE) are responsible for approximately 13 100 infections per year in the USA and are considered an urgent public health threat by the Centers for Disease Control and Prevention (CDC) [1, 2]. Mortality and healthcare costs associated with these infections are high [3, 4]. The molecular epidemiology of CRE differs by country and region [5, 6]. For example, the *Klebsiella pneumoniae* carbapenemase (KPC) is endemic in some regions of the USA, whereas the New Delhi metallo-β-lactamase (NDM) is endemic in regions of the Indian subcontinent [5, 6]. In the early stages of the CRE epidemic in the USA, KPC-producing *K. pneumoniae* ST258 was identified as the dominant strain [7, 8]. Over time, the molecular epidemiology has diversified with multiple species, multiple strains and multiple carbapenemase genes contributing to the carbapenem resistance landscape [9, 10]. For example, detection of NDM has become more common in the Antibiotic Resistance Laboratory Network in the USA [11].

Genomic sequencing is a critical tool in the investigation of CRE [12]. In addition to understanding molecular epidemiology, it can be used in outbreak investigations [12–17], and to identify the presence of genes of interest (e.g. known and putative carbapenemase genes [18, 19] or hypervirulence markers [20]) in a bacterial isolate. Several investigators have also evaluated the correlation between antimicrobial resistance genes detected by genomic sequencing and antimicrobial susceptibility testing (AST) data [21–23]. This is foundational work for understanding the potential for genomic results to provide clinically actionable data, although the use of genomic sequencing for this purpose remains aspirational currently due to the complex relationship between genotype and phenotype, cost, and turnaround time [24]. Similarly, virulence factors have been correlated with infection severity to provide new insights into why some infections lead to more severe disease than others [25].

There have been many studies performed describing the molecular epidemiology of CRE in the USA, all involving different time periods and different regions [8–11, 18, 22, 26–30]. However, few studies have incorporated the molecular epidemiology of contemporaneous and geographically matched carbapenem-susceptible *Enterobacterales* (CSE). The inclusion of these isolates allows for exploration of the ways in which carbapenem resistance develops (i.e. common strains acquiring carbapenem-resistance genes or new strains harbouring these genes). Through the Division of Healthcare Quality Promotion (DHQP) Sentinel Surveillance system, we collected CRE and CSE isolates from nine clinical laboratories in the USA from 2013 to 2016. Isolates underwent AST and genomic sequencing. We use these data to describe the molecular epidemiology of CRE and CSE at these sites during this time, to evaluate the presence of antimicrobial resistance genes, and to correlate the observed AST phenotype with the genotype for the carbapenems. We also evaluate the presence of virulence genes in the *K. pneumoniae* complex isolates.

## Methods

### Source of isolates

From October 2013 to June 2016, isolates were collected from nine clinical laboratories across the USA as part of the DHQP Sentinel Surveillance system and sent to CDC. In addition to the isolates, accompanying information included specimen collection date, patient age, specimen source, organism identification and phenotype (CRE or CSE). Participating laboratories were located in California (participated from October 2013 to September 2015), Iowa, Maryland, New Mexico, New York (two laboratories), North Carolina, Pennsylvania and Washington. All were located in metropolitan areas and seven (77.8 %) were affiliated with an academic medical centre. Each site was asked to submit approximately 12 CSE isolates every 3 months (range 10–15 per site every 3 months, *n*=1160 total) and all their CRE isolates (range 0–24 per site every 3 months, *n*=514 total). *Enterobacterales* isolates were considered CRE if they tested resistant to any carbapenem (doripenem, ertapenem, imipenem or meropenem) according to Clinical and Laboratory Standards Institute (CLSI) breakpoints or were documented to produce a carbapenemase at the clinical laboratory [31–33]. For *Morganella morganii *, *Proteus* spp. or *Providencia* spp. to be classified as CRE, they needed to test resistant to doripenem, ertapenem or meropenem as these species have intrinsically elevated imipenem minimum inhibitory concentrations (MICs) [31]. Sites were asked to submit only one isolate per patient per 3 month period for both CRE and CSE. Isolates could be from any source (sterile or non-sterile) but included isolates must have undergone identification and AST as part of the clinical laboratory’s routine workflow for clinical purposes. No isolates were collected exclusively for the purposes of this study.

### Isolate characterization at CDC

At CDC, the organism identity was confirmed with MALDI-ToF MS (Biotyper 3.1 MALDI System; Bruker Daltonics) using the Bruker and CDC MicrobeNet (https://microbenet.cdc.gov) databases; species-level identification was used for scores >2.0. AST was performed using in-house-developed frozen reference broth microdilution panels according to CLSI guidance [34]. A list of all the antimicrobials tested can be found in File S1. All categorical interpretations were determined according to CLSI guidance in the M100-S30 document [35]. For tigecycline, where no CLSI guidance was available, U.S. Food and Drug Administration (FDA) interpretive criteria were used [36]. *P-*values for comparisons of categorical variables were calculated using the chi-squared test.

### Genomic sequencing

A subset of CRE and CSE were selected for genomic sequencing including 421 CRE and 401 CSE. CRE were selected if not already sequenced for other purposes. CSE were selected so the species distribution for CSE would be similar to that of CRE. Only isolates that conformed to the phenotype to which they were submitted were eligible for genomic sequencing. At the Translational Genomics Research Institute North (TGen North), genomic DNA was extracted using the DNeasy Blood and Tissue Kit (Qiagen) following the manufacturer’s instructions for Gram-negative bacteria. Genomic libraries were prepared with a 500 bp insert size using the KAPA Library Preparation Kit (Roche) and quantified using the KAPA Library Quantification Kit (Roche) and sequenced on a MiSeq System using v2 (2×250) or v3 (2×300) chemistry (Illumina) or a NextSeq System using a High- or Mid-Output kit (2×150) (Illumina).

### Bioinformatics analysis

Genomic sequencing reads were processed through CDC’s in-house QuAISAR-H pipeline; all publicly available tools and versions can be found on the QuAISAR-H repository [37]. Specifically, sequences were processed with BBDuk v38.26 and Trimmomatic v0.35 to remove adapters and PhiX, and to quality trim sequences [38, 39]. Samples were assembled *de novo* using SPAdes v3.13.0 with settings --careful and --only-assembler and --phred-offset 33 [40]. After assembly, scaffolds of <500 bp were removed. We filtered samples on quality control metrics requiring >25× raw read coverage, <300 contigs after trimming, and the ratio of assembly length to the calculated median National Center for Biotechnology Information (NCBI) RefSeq genome size between +0.20 and −0.20. Filtering removed 125 samples from further analysis. Taxonomic identification was completed by finding the highest average nucleotide identity (ANI) against NCBI’s RefSeq database using pyANI v.0.2.11. Samples with incongruous matches between their best taxonomic hit via ANI and MALDI-ToF were also excluded (*n*=17).

Sequence types (STs) were assigned using the PubMLST database and mlst v2.19 [41, 42]. ST diversity was measured by calculating Simpson’s diversity index (1-D). 1-D ranges from 0 to 1 with higher values indicating higher diversity.

Antimicrobial resistance genes were identified using SRST2 v0.2.0 and GAMMA v2.1 against a non-redundant combined database of the antimicrobial resistance databases ResFinder, ARG-ANNOT v6 and NCBI’s AMRFinderPlus (all accessed 5 July 2021); a minimum of 98 % sequence identity and 90 % sequence coverage was required [43–47]. Samples were also searched for plasmid replicons using PlasmidFinder v1.3 [48] and for hypervirulence-associated genes (*iroB, iucA, rmpA, rmpA2* and *peg-344*) with GAMMA v2.1 in *K. pneumoniae* complex isolates [46, 49]. *K. pneumoniae* complex isolates with three or more hypervirulence-associated genes were assessed for predicted capsular serotype using Kaptive, as these polysaccharide capsules are important for *K. pneumoniae* virulence [50].

For phylogenetic analyses based on SNPs, TGen North used NASP to create an SNP matrix from the sample sets of interest [51]. First, sequence reads were aligned with BWA-MEM to a reference genome (* Escherichia coli* ST131: NCBI GenBank accession no. HG941718, *K. pneumoniae* ST258: GenBank accession no. CP006923) that had regions of recombination removed [52–55]. SNPs were called with GATK [56]. Nucleotide positions filtered out included SNP loci with <10× coverage or <90 % consensus in any one sample, and SNP loci that were not present in all the genomes in the sample set. From NASP, results were output in an SNP matrix from a core genome common to all the isolates in the analysis. Phylogenetic trees were generated from the NASP SNP matrices with mega11 and visualized in MicroReact [57, 58].

Sequence reads were submitted to NCBI’s Sequence Read Archive (SRA) database under the BioProject PRJNA288601 (File S1).

## Results

### Description of data set

Of 1160 isolates submitted as CSE and 514 isolates submitted as CRE, 1114 were confirmed as CSE (96.0 %) and 422 were confirmed as CRE (82.1 %) (Table 1). The most common CSE submitted were *Escherichia coli* (*n*=540, 46.6 %), *Klebsiella pneumoniae* complex (*n*=260, 22.4 %), other species (*n*=221, 19.1 %), *Enterobacter cloacae* complex (*n*=72, 6.2 %), *Klebsiella aerogenes* (*n*=40, 3.4 %) and *Citrobacter freundii* complex (*n*=27, 2.3 %). The most common CRE submitted were *K. pneumoniae* complex (*n*=323, 62.8%), *Enterobacter cloacae* complex (*n*=71, 13.8 %), *Escherichia coli* (*n*=60, 11.7 %), other species (*n*=27, 5.3 %), *K. aerogenes* (*n*=25, 4.9 %) and *C. freundii* complex (*n*=8, 1.6 %). Specimen sources included urine, blood, respiratory and other and varied by phenotype (Table 1). Isolates not confirmed as CSE or CRE were not submitted for genomic sequencing. Of 401 CSE isolates and 421 CRE isolates selected for genomic sequencing, the quality of sequenced isolates was acceptable for 328 CSE (81.8 %) and 352 CRE (83.6 %) (Table 1, File S2, Tables S1 and S2). Of these 680 isolates, there were 476 *K *. *pneumoniae* complex, 87 *Escherichia coli *, 71 *Enterobacter cloacae* complex, 26 *K *. *aerogenes *, 16 *C *. *freundii* complex, 1 *Klebsiella oxytoca *, 1 *Serratia marcescens *, 1 *Serratia ureilytica *, and 1 *Providencia stuartii *.

**Table 1.** Number of isolates confirmed

	Confirmed CSE (*n*=1114)	Confirmed CRE (*n*=422)
**Year collected**		
2013	102 (9.2 %)	52 (12.3 %)
2014	417 (37.4 %)	190 (45.0 %)
2015	409 (36.7 %)	126 (29.9 %)
2016	186 (16.7 %)	54 (12.8 %)
**Source**		
Blood	329 (29.5 %)	61 (14.5 %)
Respiratory	101 (9.1 %)	74 (17.5 %)
Urine	491 (44.1 %)	180 (42.7 %)
Other	193 (17.3 %)	107 (25.4 %)
**Site**		
CA	92 (8.3 %)	39 (9.2 %)
IA	127 (11.4 %)	12 (2.8 %)
MD	129 (11.6 %)	76 (18.0 %)
NM	133 (11.9 %)	16 (3.8 %)
NY1	131 (11.8 %)	113 (26.8 %)
NY2	123 (11.0 %)	35 (8.3 %)
NC	128 (11.5 %)	29 (6.9 %)
PA	124 (11.1 %)	93 (22.0 %)
WA	127 (11.4 %)	9 (2.1 %)
**Organism identity**		
* C. freundii* complex	27 (2.4 %)	8 (1.9 %)
* Enterobacter cloacae* complex	67 (6.0 %)	47 (11.1 %)
* Escherichia coli *	536 (48.1 %)	36 (8.5 %)
* K. aerogenes *	40 (3.6 %)	15 (3.6 %)
* K. pneumoniae* complex	257 (23.1 %)	307 (72.7 %)
Other	187 (16.8 %)	9 (2.1 %)
**Patient age (years, median)**	61.0	66.5

CA, California; CRE, carbapenem-resistant *Enterobacterales* CSE, carbapenem-susceptible *Enterobacterales *; IA, Iowa; MD, Maryland; NC, North Carolina; NY1, New York site #1; NY2, New York site #2; PA, Pennsylvania; WA, Washington.

**Table 2.** Number of isolates harbouring various β-lactamase genes, stratified by species and phenotype

	Carbapenemase gene	ESBL gene	AmpC gene	ESBL or AmpC gene
***C. freundii*** **complex (** ***n*** **=16)**	7 (43.8 %)	1 (6.3 %)	16 (100 %)	16 (100 %)
CP-CRE (*n*=7)	7 (100 %)	1 (14.3 %)	7 (100 %)	7 (100 %)
Non-CP-CRE (*n*=0)	na	na	na	na
CSE (*n*=9)	0 (0 %)	0 (0 %)	9 (100 %)	9 (100 %)
***Enterobacter cloacae*** **complex (** ***n*** **=71)**	18 (25.4 %)	15 (21.1 %)	68 (95.8 %)	69 (97.2 %)
CP-CRE (*n*=18)	18 (100 %)	9 (50.0 %)	17 (94.4 %)	18 (100 %)
Non-CP-CRE (*n*=19)	na	4 (21.1 %)	18 (94.7 %)	18 (94.7 %)
CSE (*n*=34)	0 (0 %)	2 (5.9 %)	33 (97.1 %)	33 (97.1 %)
***Escherichia coli*** **(** ***n*** **=87)**	16 (18.4 %)	28 (32.2 %)	8 (9.2 %)	34 (39.1 %)
CP-CRE (*n*=16)	16 (100 %)	5 (31.3 %)	0 (0 %)	5 (31.3 %)
Non-CP-CRE (*n*=15)	na	10(66.7 %)	7(46.7 %)	15 (100 %)
CSE (*n*=56)	0 (0 %)	13(23.2 %)	1 (1.8 %)	14 (25.0 %)
***K. aerogenes*** **(** ***n*** **=26)**	1 (3.8 %)	0 (0 %)	22 (84.6 %)	22 (84.6 %)
CP-CRE (*n*=1)	1 (100 %)	0 (0 %)	1 (100 %)	1 (100 %)
Non-CP-CRE (*n*=11)	na	0 (0 %)	8 (72.7 %)	8 (72.7 %)
CSE (*n*=14)	0 (0 %)	0 (0 %)	13 (92.9 %)	13 (92.9 %)
***K. pneumoniae*** **complex (** ***n*** **=476)**	239 (50.2 %)	129 (27.1 %)	5 (1.1 %)	134(28.2 %)
CP-CRE (*n*=238)	238 (100 %)	63 (26.5 %)	5 (2.1 %)	68 (28.6 %)
Non-CP-CRE (*n*=23)	na	16 (69.6 %)	0 (0 %)	16 (69.6 %)
CSE (*n*=215)	1 (0.5 %)	50 (23.3 %)	0 (0 %)	50 (23.3 %)
**All isolates (** ***n*** **=680)**	283 (41.6 %)	175 (25.7 %)	121 (17.8 %)	279 (41.0 %)
CP-CRE (*n*=282)	282 (100 %)	78 (27.7 %)	32 (11.3 %)	101 (35.8 %)
Non-CP-CRE (*n*=70)	na	32 (45.7 %)	33 (47.1 %)	59 (84.3 %)
CSE (*n*=328)	1 (0.3 %)	65 (19.8 %)	56 (17.1 %)	119 (36.3 %)

Carbapenemase genes include *bla*_IMP_, *bla*_KPC_, *bla*_NDM_, *bla*_OXA-48_, *bla*_OXA-181_ and *bla*_SME_.

ESBL genes include *bla*_CTX-M_, *bla*_OXA-2_, *bla*_OXA-10_, *bla*_OXY-1-3_, *bla*_SHV-2_, *bla*_SHV-2a_, *bla*_SHV-7_, *bla*_SHV-12_, *bla*_SHV-27_, *bla*_SHV-38_, *bla*_SHV-40_, *bla*_SHV-45_ and *bla*_TEM-12_.

AmpC genes include *bla*_ACT_, *bla*_CMH_, *bla*_CMY_, *bla*_MIR_, *bla*_SRT_ and cmy2-mir-act-ec (the *Klebsiella aerogenes* AmpC gene).

CP-CRE, carbapenemase-producing carbapenem-resistant *Enterobacterales *; CSE, carbapenem-susceptible *Enterobacterales *; ESBL, extended-spectrum β-lactamase Non-CP-CRE, non-carbapenemase-producing carbapenem-resistant *Enterobacterales *.

### CDC AST data and presence of β-lactamase genes

Among 352 confirmed CRE with quality sequencing data, 47 were resistant to only one carbapenem (13.4 %), 305 were resistant to two or more carbapenems (86.6 %), and 263 were resistant to all carbapenems tested (74.7 %). Of the 47 isolates resistant to only one carbapenem, 46 were resistant to ertapenem and one was resistant to imipenem. Overall, 282 CRE isolates harboured a carbapenemase gene (80.1 %); 15 were resistant to only one carbapenem (5.3 %), 267 were resistant to two or more carbapenems (94.7 %), and 244 were resistant to all carbapenems tested (86.5 %). There were 276 CRE isolates with *bla*_KPC_; 13 were resistant to only one carbapenem (4.7 %), 263 were resistant to two or more carbapenems (95.3 %), and 241 were resistant to all carbapenems tested (87.3 %). No isolates harboured more than one carbapenemase. There were 70 CRE isolates without a carbapenemase (19.9 %); 32 were resistant to only one carbapenem (45.7 %), 38 were resistant to two or more carbapenems (54.3 %), and 19 were resistant to all carbapenems tested (27.1 %).

**Fig. 1.**
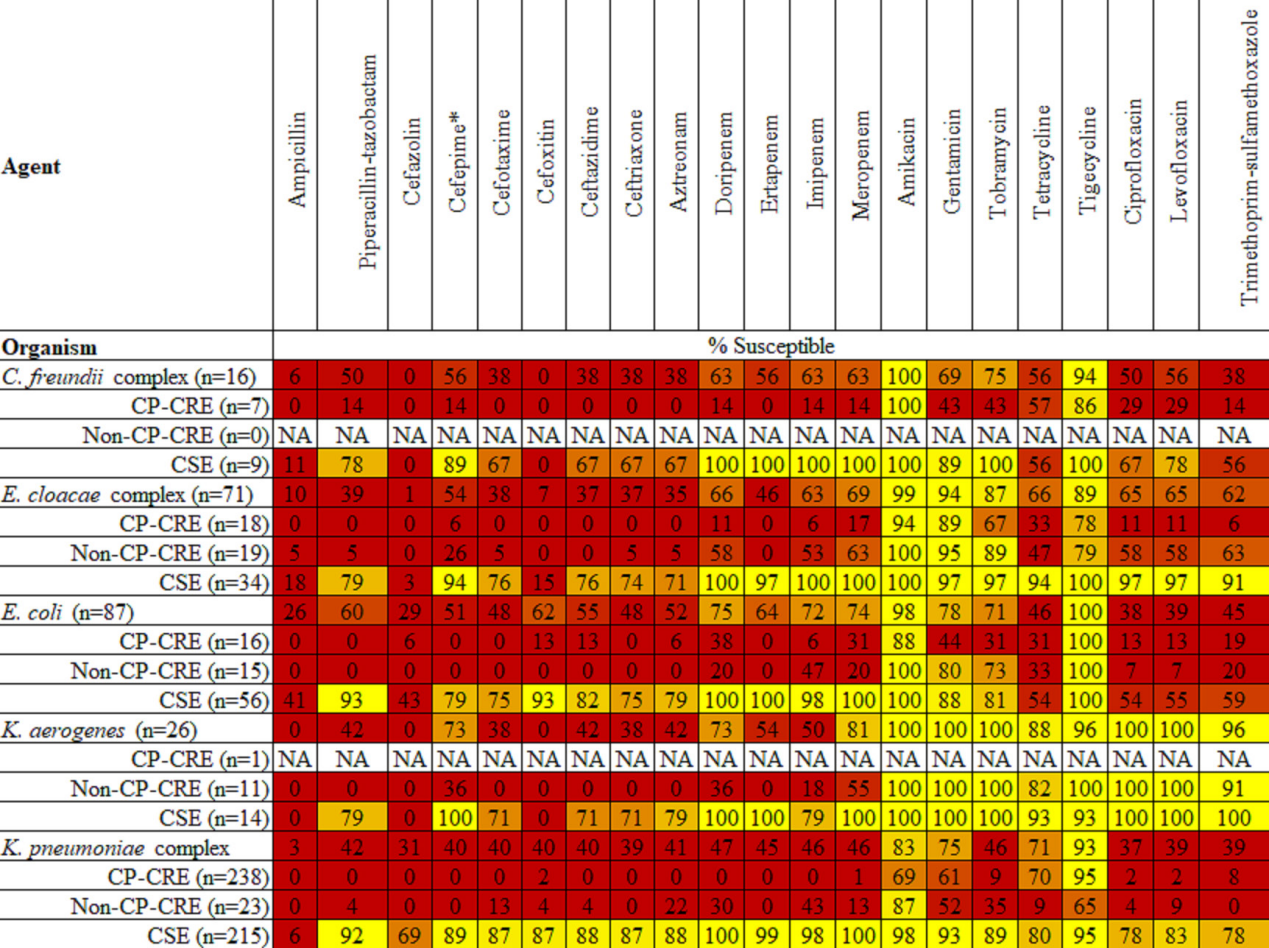
AST data for *Citrobacter freundii* complex, *Enterobacter cloacae* complex, *Escherichia coli *, *Klebsiella aerogenes* and *Klebsiella pneumoniae* complex stratified by phenotype (% Susceptible). Values of 90–100 % are yellow, 0–50 % red and values of 51–89 % are shades of orange (yellow to red). CP-CRE: carbapenemase-producing carbapenem-resistant *Enterobacterales *, Non-CP-CRE: non-carbapenemase-producing carbapenem-resistant *Enterobacterales *, CSE: carbapenem-susceptible *Enterobacterales *. Data are only provided for phenotypes with *n*≥5. *This does not include susceptible dose-dependent isolates.

In addition to the four carbapenems, AST data were generated for 19 additional antimicrobial agents (Fig. 1, File S1). These data were also stratified by carbapenemase producing (CP)-CRE status (CP-CRE, non-CP-CRE or CSE). For some non-beta-lactam bug–drug combinations, CP-CRE were less frequently susceptible to antimicrobial agents than non-CP-CRE. An example of this pattern is ciprofloxacin and *Enterobacter cloacae* complex; 11 % of CP-CR *Enterobacter cloacae* complex were susceptible to ciprofloxacin compared to 58 % of non-CP-CR *Enterobacter cloacae* complex (*P*=0.0029). Another example is tobramycin and *Escherichia coli *; 31 % of CP-CR *Escherichia coli* were susceptible to tobramycin compared to 73 % of non-CP-CR *Escherichia coli* (*P*=0.0191).

In addition, the frequency of various β-lactamase genes was analysed for each category of isolate (CP-CRE, non-CP-CRE and CSE) and species (Table 2). For *Enterobacter cloacae* complex, extended-spectrum beta-lactamase (ESBL) genes appeared more frequently in CP-CRE (50.0 %) than in non-CP-CRE (21.1 %), although this was not statistically significant (*P*=0.0653). For *Escherichia coli *, ESBL genes were more frequent in non-CP-CRE (66.7 %) than in CP-CRE (31.3%) (*P*=0.0486). Similarly for *K. pneumoniae* complex, ESBL genes were more frequent in non-CP-CRE (69.6 %) than in CP-CRE (26.5 %) (*P*<0.0001). AmpC genes were present in 46.7 % of non-CP-CR *Escherichia coli* isolates, and were rare in *K *. *pneumoniae* complex isolates (1.1 %).

### Molecular epidemiology of the isolates

**Fig. 2.**
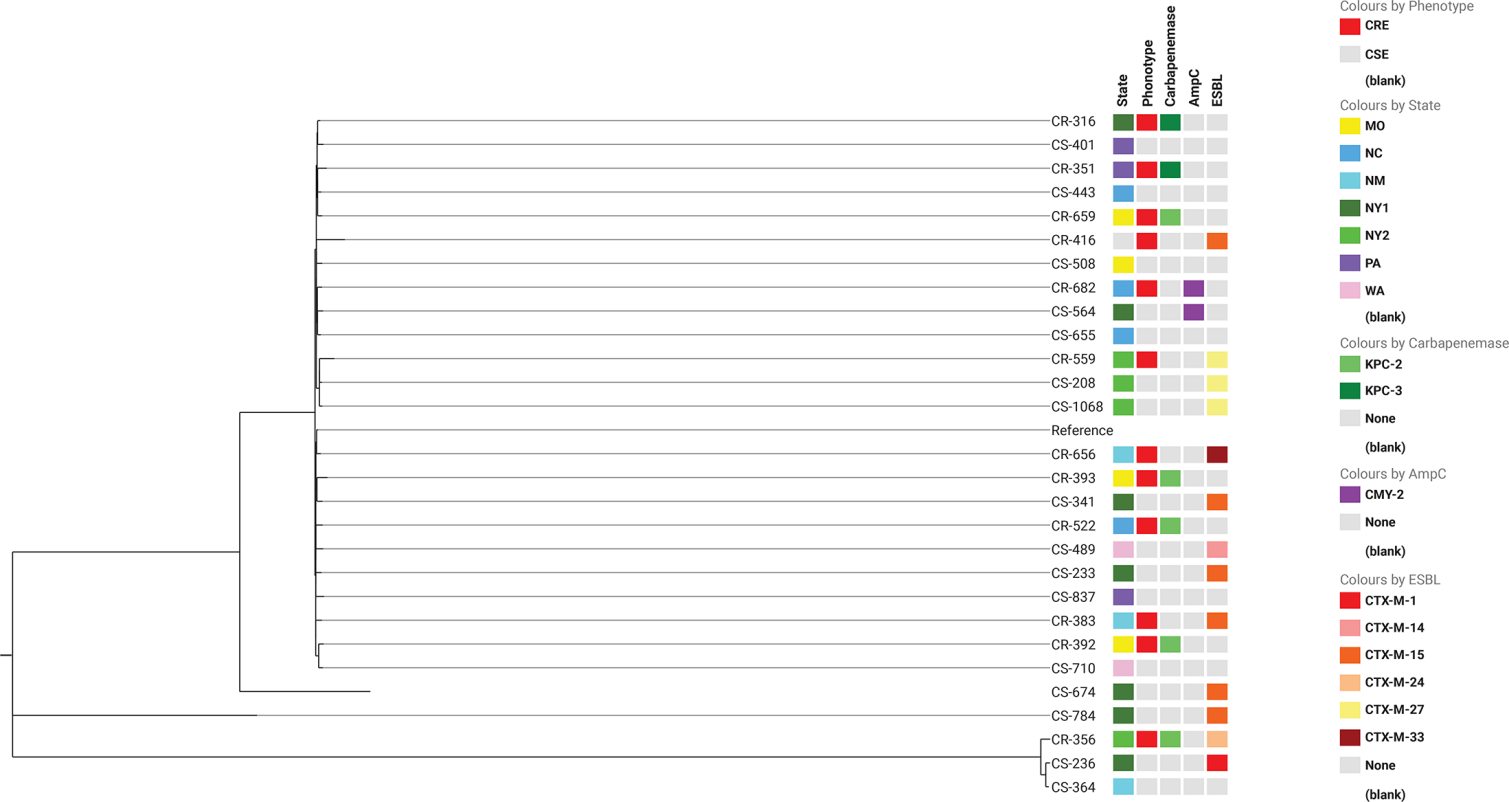
Maximum-parsimony phylogenetic tree (with 10 bootstrap replicates) based on 13 297 parsimony-informative SNPs in 28 genomes of *Escherichia coli* ST131 isolates aligned to the EC958 genome (GenBank accession no. HG941718) with two apparent regions of recombination removed (positions 3385000–3420000 and 3425000–3455000). This analysis covered 4.17 Mb (82.6 % of the 4.81 Mb reference genome of EC958). The coverage for each genome in the dataset ranged from 88.8 to 94.6 % of the reference genome. The consistency index is 0.95, indicating a low level of homoplasy in the dataset. The figure can be found at: https://microreact.org/project/kddx7Bby9VYhM1iVAQ3CQJ-st131-cdc.

Simpson’s diversity index (1-D) values for each species and phenotype revealed high diversity with the exception of CR *K. pneumoniae* complex (1-D=0.38) (Table 3). There were 164 unique STs for the 476 *K *. *pneumoniae* complex isolates (Table 4, File S2, Fig. S1A). There was also one isolate for which an ST could not be generated due to an incomplete sequence for a single gene. The most common ST was ST258 (*n*=207) comprising 43.5 % of all isolates; 98.6 % of ST258 isolates harboured *bla*_KPC_ (File S2, Fig. S2). There were 39 unique STs for the 87 *Escherichia coli* isolates (Table 4, File S2, Fig. S1B). ST131 was most common and accounted for 32.2 % of all *Escherichia coli* (*n*=28) (Fig. 2). There were 47 unique STs for the 71 *Enterobacter cloacae* complex isolates (Table 4, File S2, Fig. S1C).

**Table 3.** Simpson’s diversity index (1-D) by phenotype and species

	Simpson’s diversity index (1-D)
**CRE**	
* Citrobacter freundii* complex (*n*=7)	0.95
* Enterobacter cloacae* complex (*n*=37)	0.97
* Escherichia coli* (*n*=31)	0.84
* Klebsiella aerogenes* (*n*=12)	0.91
* Klebsiella pneumoniae* complex (*n*=261)	0.38
**CSE**	
* Citrobacter freundii* complex (*n*=9)	1.00
* Enterobacter cloacae* complex (*n*=34)	0.97
* Escherichia coli* (*n*=56)	0.91
* Klebsiella aerogenes* (*n*=14)	0.89
* Klebsiella pneumoniae* complex (*n*=215)	0.99

1-D ranges from 0 to 1 with higher values indicating higher diversity.

**Table 4.** Molecular epidemiology, carbapenemase genes and sequence types

Organism	No. of unique STs	Carbapenemase genes (*n*)	CP-CRE STs (*n*)	Non-CP-CRE STs (*n*)	CSE STs (*n*)
* C. freundii* complex (*n*=16)	14	KPC-2 (6), KPC-3 (1)	ST22 (2), other (5)		ST22 (1), other (8)
* Enterobacter cloacae* complex (*n*=71)	47	KPC-3 (13), KPC-2 (4), KPC-4 (1)	ST114 (6), ST171 (3), ST145 (1), other (8)	ST32 (2), ST116 (2), ST50 (1), other (14)	ST50 (6), ST145 (2), ST32 (1), ST116 (1), other (24)
* Escherichia coli* (*n*=87)	39	KPC-2 (7), KPC-3 (5), OXA-181 (2), NDM-5 (1), NDM-7 (1)	ST131 (7), ST38 (1), ST69 (1), ST405 (1), other (6)	ST131 (5), ST405 (3), ST648 (3), other (4)	ST131 (16), ST73 (4), ST1193 (4), ST95 (3), ST127 (3), ST38 (2), ST69 (2), ST648 (1), other (21)
* K. aerogenes* (*n*=26)	15	KPC-2 (1)	Other (1)	ST93 (4), other (7)	ST93 (5), other (9)
* K. pneumoniae* complex (*n*=476)	164	KPC-3 (156), KPC-2 (79), KPC-4 (1), KPC-34 (1), OXA-48 (1), IMP-8 (1)	ST258 (204), ST48 (4), ST17 (3), ST340 (3), ST11 (2), ST15 (2), ST392 (2), ST34 (1), ST39 (1), ST76 (1), ST234 (1), ST307 (1)	ST307 (3), ST15 (2), ST17 (2), ST45 (2), ST258 (2), ST11 (1), ST101 (1), ST392 (1)	ST45 (10), ST17 (9), ST20 (7), ST15 (6), ST11 (5), ST253 (5), ST35 (4), ST14 (3), ST16 (3), ST23 (3), ST29 (3), ST37 (3), ST234 (3), ST433 (3), ST34 (2), ST39 (2), ST76 (2), ST101 (2), ST392 (2), ST258 (1), ST307 (1)

*Other includes STs with *n*≤2.

Plamsid markers were identified in 589 of 680 isolates (86.6 %). CRE isolates carried a mean of 3.3 plasmid markers and CSE carried a mean of 1.9 markers. A detailed list of identified plasmid markers can be found in File S1.

We identified six *K *. *pneumoniae* complex isolates with three or more hypervirulence-associated genes (File S1). None of the six were carrying a carbapenemase gene (all were CSE). One isolate, an ST86, had three of five hypervirulence-associated genes evaluated here (*iroB, peg-344* and *rmpA*). Two isolates were ST380 and predicted serotype K2, and carried four hypervirulence-associated genes (*iroB, iucA, rmpA* and *peg-344*). Three ST23, K1 serotype (predicted) isolates carried all five hypervirulence-associated genes (*iroB, iucA, rmpA, rmpA2* and *peg-344*).

## Discussion

This study provides a snapshot of the molecular epidemiology of CRE at nine sites in the USA between 2013 and 2016. A unique feature was the inclusion of CSE. We describe the predominance of ST258 amongst the *K. pneumoniae* complex isolates with *bla*_KPC_ (86.1%) and the predominance of ST131 amongst both CR (38.7%) and CS (28.6%) *Escherichia coli* isolates (32.2 % overall). This confirms what others have found over the same period [18]. ST131 *Escherichia coli* isolates did not cluster by site or phenotype (CRE vs. CSE). In contrast, the ST258 *K. pneumoniae* complex isolates clustered by site. These findings along with the Simpson’s diversity index results suggest that CR in the *K. pneumoniae* complex is driven by clonal transmission.

Inclusion of CSE also allows for monitoring of potential reservoirs of hypervirulent *Klebsiella *. We identified biomarkers for hypervirulence in six *K *. *pneumoniae* complex isolates without carbapenemase genes. These isolates were identified in combinations of serotype and ST known for hypervirulence [59–62] and all carried the plasmid-mediated *rmpA* associated with a mucoid phenotype [63]. There is the risk that these STs associated with hypervirulence may acquire carbapenemases, which has already been documented [20].

This study has detailed AST data using reference broth microdilution which can be analysed in conjunction with genomic sequencing data. We focused on carbapenemase genes and the CR phenotype and found 80.1 % of the CRE isolates harboured carbapenemase genes, although this varied depending on the site (range: 9.1–96.3 %) and species (only 8.3 % of CR *K. aerogenes* had a carbapenemase while 100 % of CR *C. freundii* complex had a carbapenemase). Other CDC studies using different catchment areas and periods have found that 35–73 % of CRE are CP-CRE [18, 29, 64]. The percentage was probably higher in this study given the geographical distribution of sites submitting isolates. For example, there were two sites from New York, and the early impact of the KPC outbreak in New York has been well described [65–67]. Most carbapenemase genes detected in this study were *bla*_KPC_ variants (97.5%), which is consistent with the molecular epidemiology of CRE at the time the study was conducted [18]. One unique finding was a *bla*_IMP-8_ isolate (sample name: CS-666) that was submitted as CSE and confirmed as carbapenem-susceptible. This isolate tested as intermediate to all four carbapenems. This could be an instance of a minor AST error (labelling an isolate as intermediate when it is resistant) as broth microdilution MIC testing is only accurate to within ±1 doubling dilution [68]. Another possible explanation is that *bla*_IMP-8_ is a weak carbapenemase and isolates with this gene frequently test susceptible to carbapenems [69]. In addition to the CR phenotypes, many other genotype–phenotype associations can be examined as there are data on aminoglycosides, fluoroquinolones and many other drugs.

This work is subject to several limitations. Most significantly, the isolates were collected in 2013–2016, so the molecular epidemiology at the time this study was conducted may not reflect the current epidemiology. For example, CP-CRE was more frequently due to *bla*_KPC_ at the time the study was conducted. Also, the sites may not be representative of the USA as a whole. All laboratories submitting isolates for this study were in metropolitan areas and most were affiliated with academic medical centres. Also, the isolates were tested several years ago so newer antimicrobial agents (e.g. ceftazidime-avibactam, meropenem-vaborbactam, imipenem-relebactam and cefiderocol) were not included and the modified carbapenem inactivation method test was not performed to evaluate the presence of phenotypic carbapenemase production. Furthermore, only ~80 % of the isolates were successfully sequenced, which is a high failure rate, but we do not think there was a bias in which isolates failed sequencing. We also did not look at porin mutations or other mutations that may have contributed to carbapenem resistance beyond the presence of antimicrobial resistance genes. Finally, the isolates were submitted as a convenience sample rather than a random sample, so the isolates sent to CDC may be biased in some way. For example, a greater proportion of CSE were blood isolates compared to CRE.

This unique dataset of 680 isolates significantly bolsters publicly available data for public health, molecular epidemiology and molecular biology efforts. It comprises high-quality genomic data, not only from verified CRE but also from CSE, from across the USA. We believe this dataset serves as a significant resource for further analyses as the genomic data are curated with each isolate’s AST data. In addition, these data may be valuable for improving the understanding of phylogenomics, the spread of carbapenem resistance and molecular epidemiology. Continued efforts not only to generate and share microbiological data but also to expand, fully mine and harness these data are critical, as these scientific findings have and will continue to provide tools for public health action and, in some cases, clinical decision-making.

## Supplementary Data

Supplementary material 1

Supplementary material 2
