# Supplementary material for: Comparison of carbapenem-susceptible and carbapenem-resistant Enterobacterales at nine sites in the USA, 2013–2016: a resource for antimicrobial resistance investigators
Source: Microb Genom. 2023 Nov 21;9(11):001119. doi: 10.1099/mgen.0.001119 (PMC10711308; doi:10.1099/mgen.0.001119)
Supplement: Supplementary material 2 [file mgen-9-1119-s002.pdf]

Supplemental File 2, Table 1: Number of isolates submitted, confirmed, sequenced, and whose quality was acceptable

	Submitted CSE (n=1160)	Confirmed CSE (n=1114)	Sequenced CSE (n=401)	Quality Sequenced CSE (n=328)	Submitted CRE (n=514)	Confirmed CRE (n=422)	Sequenced CRE (n=421)	Quality Sequenced CRE (n=352)
Year Collected								
2013	106 (9.1%)	102 (9.2%)	35 (8.7%)	27 (8.2%)	57 (11.1%)	52 (12.3%)	52 (12.4%)	43 (12.2%)
2014	439 (37.8%)	417 (37.4%)	137 (34.2%)	98 (29.9%)	224 (43.6%)	190 (45.0%)	189 (44.9%)	151 (42.9%)
2015	423 (36.5%)	409 (36.7%)	162 (40.4%)	142 (43.3%)	158 (30.7%)	126 (29.9%)	126 (29.9%)	114 (32.4%)
2016	192 (16.6%)	186 (16.7%)	67 (16.7%)	61 (18.6%)	75 (14.6%)	54 (12.8%)	54 (12.8%)	44 (12.5%)
Source								
Blood	344 (29.7%)	329 (29.5%)	149 (37.2%)	115 (35.1%)	74 (14.4%)	61 (14.5%)	61 (14.5%)	51 (14.5%)
Respiratory	105 (9.1%)	101 (9.1%)	33 (8.2%)	28 (8.5%)	84 (16.3%)	74 (17.5%)	74 (17.6%)	65 (18.5%)
Urine	507 (43.7%)	491 (44.1%)	156 (38.9%)	131 (39.9%)	225 (43.8%)	180 (42.7%)	179 (42.5%)	153 (43.5%)
Other	204 (17.6%)	193 (17.3%)	63 (15.7%)	54 (16.5%)	131 (25.5%)	107 (25.4%)	107 (25.4%)	83 (23.6%)
Site								
CA	97 (8.4%)	92 (8.3%)	36 (9.0%)	29 (8.8%)	44 (8.6%)	39 (9.2%)	39 (9.3%)	37 (10.5%)
IA	132 (11.4%	127 (11.4%)	26 (6.5%)	18 (5.5%)	15 (2.9%)	12 (2.8%)	12 (2.9%)	10 (2.8%)
MD	132 (11.4%)	129 (11.6%)	56 (14.0%)	46 (14.0%)	96 (18.7%)	76 (18.0%)	76 (18.1%)	63 (17.9%)
NM	137 (11.8%)	133 (11.9%)	42 (10.5%)	36 (11.0%)	45 (8.8%)	16 (3.8%)	16 (3.8%)	11 (3.1%)
NY1	132 (11.4%)	131 (11.8%)	48 (12.0%)	42 (12.8%)	125 (24.3%)	113 (26.8%)	113 (26.8%)	90 (25.6%)
NY2	132 (11.4%)	123 (11.0%)	64 (16.0%)	46 (14.0%)	50 (9.7%)	35 (8.3%)	35 (8.3%)	28 (8.0%)
NC	133 (11.5%)	128 (11.5%)	50 (12.5%)	42 (12.8%)	36 (7.0%)	29 (6.9%)	28 (6.7%)	25 (7.1%)
PA	130 (11.2%)	124 (11.1%)	42 (10.5%)	36 (11.0%)	94 (18.3%)	93 (22.0%)	93 (22.1%)	82 (23.3%)
WA	135 (11.6%)	127 (11.4%)	37 (9.2%)	33 (10.1%)	9 (1.8%)	9 (2.1%)	9 (2.1%)	6 (1.7%)
Organism Identity								
C. freundii complex	27 (2.3%)	27 (2.4%)	10 (2.5%)	9 (2.7%)	8 (1.6%)	8 (1.9%)	8 (1.9%)	7 (2.0%)
E. cloacae complex	72 (6.2%)	67 (6.0%)	43 (10.7%)	34 (10.4%)	71 (13.8%)	47 (11.1%)	47 (11.2%)	37 (10.5%)
E. coli	540 (46.6%)	536 (48.1%)	70 (17.5%)	56 (17.1%)	60 (11.7%)	36 (8.5%)	35 (8.3%)	31 (8.8%)
K. aerogenes	40 (3.4%)	40 (3.6%)	22 (5.5%)	14 (4.3%)	25 (4.9%)	15 (3.6%)	15 (3.6%)	12 (3.4%)

K. pneumoniae complex	260 (22.4%)	257 (23.1%)	256 (63.8%)	215 (65.5%)	323 (62.8%)	307 (72.7%)	307 (72.9%)	261 (74.1%)
Other	221 (19.1%)	187 (16.8%)	0 (0.0%)	0 (0.0%)	27 (5.3%)	9 (2.1%)	9 (2.1%)	4 (1.1%)
Patient Age (Median)	61.0	61.0	62.0	62.0	67.0	66.5	66.5	66.0

CA: California, IA: Iowa, MD: Maryland, NY1: New York site #1, NY2: New York site #2, NC: North Carolina, PA: Pennsylvania, WA: Washington, CSE: carbapenem-susceptible Enterobacterales, CRE: carbapenem-resistant Enterobacterales

Supplemental File 2, Table 2: Phenotype and organism frequencies by site (identification based on MALDI-ToF result at CDC)

	CA	IA	MD	NM	NY1	NY2	NC	PA	WA	All
C. freundii complex	0	5	7	0	4	0	0	0	0	16
CRE	0	2	3	0	2	0	0	0	0	7
CSE	0	3	4	0	2	0	0	0	0	9
E. cloacae complex	3	2	14	10	7	15	18	0	2	71
CRE	1	1	9	3	6	8	9	0	0	37
CSE	2	1	5	7	1	7	9	0	2	34
E. coli	7	5	13	13	16	8	9	9	7	87
CRE	2	0	9	3	6	3	3	1	4	31
CSE	5	5	4	10	10	5	6	8	3	56
K. aerogenes	3	0	8	5	0	1	7	2	0	26
CRE	2	0	1	3	0	1	3	2	0	12
CSE	1	0	7	2	0	0	4	0	0	14
K. pneumoniae complex	53	15	65	19	105	50	32	107	30	476
CRE	32	6	39	2	76	16	9	79	2	261
CSE	21	9	26	17	29	34	23	28	28	215
Total*	66	28	109	47	132	74	67	118	39	680
CRE*	37	10	63	11	90	28	25	82	6	352
CSE	29	18	46	36	42	46	42	36	33	328

*In addition to the above organisms, there was 1 *Klebsiella oxytoca* (CRE) from MD, 1 *Providencia stuartii* (CRE) from NC, 1 *Serratia marcescens* (CRE) from IA, and 1 *Serratia ureilytica* (CRE) from MD.

CA: California, IA: Iowa, MD: Maryland, NY1: New York site #1, NY2: New York site #2, NC: North Carolina, PA: Pennsylvania, WA: Washington, CRE: carbapenem-resistant Enterobacterales, CSE: carbapenem-susceptible Enterobacterales

Supplemental File 2, Figure 1: The graphs illustrate the most common sequence types (STs) for *Klebsiella pneumoniae* complex (1A), *Escherichia coli* (1B), and *Enterobacter cloacae* complex (1C). Only STs with >2 isolates are included in the graphs. Note that the y-axes are of differing scales and the y-axis in 2A is broken.

CP-CRE: carbapenemase-producing carbapenem-resistant Enterobacterales, Non-CP-CRE: non-carbapenemase-producing carbapenem-resistant Enterobacterales, CSE: carbapenem-susceptible Enterobacterales

Supplemental File 2, Figure 2: Maximum parsimony phylogenetic tree (with 50 bootstrap replicates) based on 3,479 parsimony-informative SNPs in 204 CP-carbapenem-resistant *Klebsiella pneumoniae* isolates aligned to the NJST258-1 genome (Genbank accession no. CP006923) with two apparent regions of recombination removed (one previously identified and removed, and one specific to this dataset at positions 4,870,001-4,890,001). This analysis covered 3.48 Mbp (71.3% of the 4.69 Mbp reference genome). The coverage for each genome in the dataset ranged from 92.0-96.1% of the reference genome. The consistency index is 0.99 indicating a low level of homoplasy in the dataset. The figure can be found here: https://microreact.org/project/2Pr5FKFbaaj5oQx3D3mtqg-st258-cdc.

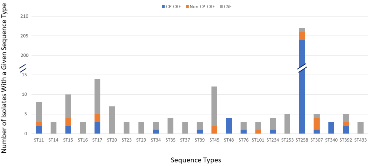

CP-CRE
Non-CP-CRE
CSE
Number of Isolates With a Given Sequence Type
210
205
200
15
10
5
0
ST11
ST14
ST15
ST16
ST17
ST20
ST23
ST29
ST34
ST35
ST37
ST39
ST45
ST48
ST76
ST101
ST234
ST253
ST258
ST307
ST340
ST392
ST433
Sequence Types

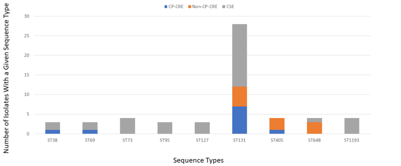
CP-CRE
Non-CP-CRE
CSE
Number of Isolates With a Given Sequence Type
0
5
10
15
20
25
30
ST38
ST69
ST73
ST95
ST127
ST131
ST405
ST648
ST1193
Sequence Types

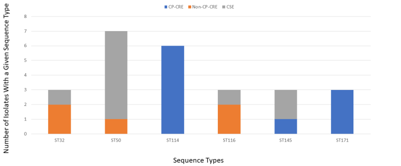
CP-CRE
Non-CP-CRE
CSE
8
7
6
5
4
3
2
1
0
ST32
ST50
ST114
ST116
ST145
ST171
Number of Isolates With a Given Sequence Type
Sequence Types

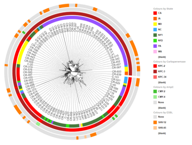

Colours by State
CA
IA
MD
NC
NY1
NY2
PA
WA
(blank)
Colours by Carbapenemase
KPC-2
KPC-3
KPC-34
(blank)
Colours by AmpC
CMY-2
CMY-4
None
(blank)
Colours by ESBL
None
SHV-12
SHV-45
(blank)
